# Ex Vivo Human Colon Tissue Exposure to Pristine Graphene Activates Genes Involved in the Binding, Adhesion and Proliferation of Epithelial Cells

**DOI:** 10.3390/ijms222111443

**Published:** 2021-10-23

**Authors:** Mohamed H. Lahiani, Kuppan Gokulan, Katherine Williams, Sangeeta Khare

**Affiliations:** Division of Microbiology, National Center for Toxicological Research, U.S. Food and Drug Administration, 3900 NCTR Rd, Jefferson, AR 72079, USA; hassen86us@gmail.com (M.H.L.); kuppan.gokulan@fda.hhs.gov (K.G.); kgmwilliams8@gmail.com (K.W.)

**Keywords:** graphene, nanomaterial, toxicity, risk assessment, nonanimal model

## Abstract

Toxicology studies on pristine graphene are limited and lack significant correlations with actual human response. The goal of the current study was to determine the response of total colonic human tissue to pristine graphene exposure. Biopsy punches of colon tissues from healthy human were used to assess the biological response after ex vivo exposure to graphene at three different concentrations (1, 10, and 100 µg/mL). mRNA expression of specific genes or intestinal cytokine abundance was assessed using real-time PCR or multiplex immunoassays, respectively. Pristine graphene-activated genes that are related to binding and adhesion (*GTPase* and *KRAS*) within 2 h of exposure. Furthermore, the *PCNA* (proliferating cell nuclear antigen) gene was upregulated after exposure to graphene at all concentrations. Ingenuity pathway analysis revealed that STAT3 and VEGF signaling pathways (known to be involved in cell proliferation and growth) were upregulated. Graphene exposure (10 µg/mL) for 24 h significantly increased levels of pro-inflammatory cytokines IFNγ, IL-8, IL-17, IL-6, IL-9, MIP-1α, and Eotaxin. Collectively, these results indicated that graphene may activate the STAT3–IL23–IL17 response axis. The findings in this study provide information on toxicity evaluation using a human-relevant ex vivo colon model and serve as a basis for further exploration of its bio-applications.

## 1. Introduction

With the rise of nanotechnology in the last decade, many nanomaterials have shown potential uses in real applications. Graphene, a single layer of monocrystalline graphite with sp^2^ hybridized carbon atoms, has attracted many researchers due to its wide utilization in technological and biomedical applications [1]. Graphene is available in pristine form or other functionalized forms. Graphene-related materials (GRMs) comprise several chemistries, for example, few-layer graphene (FLG), graphene oxide (GO), reduced graphene oxide (RGO), graphene nanosheets, graphene nanoribbons, and graphene quantum dots [2,3]. Graphene is a suitable candidate for many applications in the food industry, ranging from food packaging to agriculture [4,5,6,7,8,9]. The organic solvent nanofiltration, made with graphene oxide and other substances, has shown a reduction in membrane porosity and an increase in antibacterial activity against prokaryotic organisms, such as *Staphylococcus aureus* and *Escherichia coli* [5]. On the other hand, graphene stimulates eukaryotic cells, which results in an increase in the seed germination [10], flowering [8] and growth of many different plants [9,11,12].

Human exposure to graphene can occur through dermal, ingestion, inhalation, or intravenous routes. Although the majority of published articles have evaluated the toxicity of graphene from an occupational health perspective and have focused mostly on the impact of graphene on the respiratory system [13,14], research on the health risks of ingested graphene or other nanoparticles is limited. Recent research by our group has shown that carbon nanoparticles penetrate the cell membrane and change the gene expression profile of exposed T-84 epithelial cells [15]. These nanomaterials, if accumulated in high concentrations inside edible products (for example, tomato fruit), could lead to modulation of the intestinal microbiota composition and the gastrointestinal epithelial cell barrier [15]. According to life cycle release modeling studies, soils/sediments and landfills are the primary source for an estimated 80% of carbon-based nanomaterial released into the environment [16]. Moreover, there are critical knowledge gaps regarding the fate of such nanoparticles in soil-based investigations of human exposure under environmentally relevant conditions [17]. The mucosal barrier of the intestinal tract is among the most important biological barriers within the mammalian body. It participates in nutrient uptake, defense against pathogenic bacteria, and maintains a niche between host immune cells and commensal bacteria [18]. Pristine graphene has been found to penetrate intestinal cells and translocate within different tissues [19]. After ingestion, graphene can transit through different compartments of the gastrointestinal system. A longer retention time in the large intestine could lead to close interaction of luminal contents containing graphene with the mucosal layer in the colon, which is also correlated to bacterial metabolism and mucosal turnover [20]. Furthermore, we have previously shown that pristine graphene may change the diversity in the intestinal microbial population and also impact the levels of short-chain fatty acid [21]. The fate of nanosize graphene in human tissue remains controversial. Recent studies have found that pristine graphene sheets can be degraded by oxidative enzymes secreted by immune cells [22]. The materials can also be decomposed and absorbed by phagocytes [23]. In fact, our earlier study demonstrated the phagocytosis of pristine graphene by macrophages cultured in vitro [24]. However, graphene nanosheets can assemble into fibers and three-dimensional structures that could bio-persist and not degrade [25].

**Figure 1:**
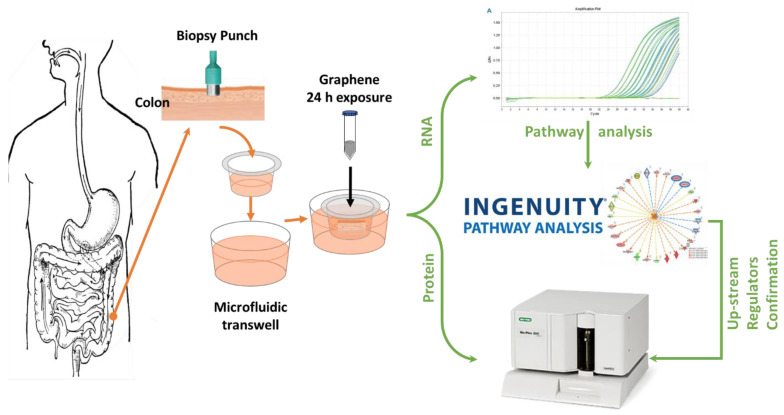
Schematic figure of experimental design.

Considering the positive impact of graphene on plant cell growth and proliferation, as well as other in other medical uses (drug delivery), it is logical to question if the longer retention time of graphene in the colon could have any impact on the growth or excessive proliferation of cells in the lining of the colon. Thus, the current study investigated the impact of graphene exposure through molecular profile (mRNA expression), as well as through levels of key cytokines/chemokines in the colonic tissue, to reveal the mechanisms by which graphene could interact with the human intestinal tract. We have previously shown that pristine graphene can affect the gastrointestinal system through modulation of the rat intestinal microbiome at doses equal to or higher than 100 µg/mL [21]. Here, the aim of this study was to evaluate the interaction of pristine graphene with the human intestinal barrier using an ex vivo model. In this study, fresh human colon tissues from different subjects were exposed to pristine graphene to identify mRNA gene expression and immune responses. The study design is shown in Figure 1. In particular, we assessed the impact of the interaction of intestinal mucosa with graphene at different doses (1, 10, and 100 µg/mL) at 2 and 24 h, with a focus on genes that are predicted to be involved in gastrointestinal regulation. The objectives in this research study were to determine: (i) the canonical pathways affected by graphene exposures to human ex vivo intestinal explants; (ii) the upstream regulators of different processes induced by graphene exposure; and (iii) the link between the immune responses activated by graphene exposure with the cellular processes also affected by graphene exposure.

## 2. Results and Discussions

This study evaluated changes in human colonic tissue gene expression and immune response after 2 and 24 h of exposure to pristine graphene. The 2 and 24 h time points were chosen to mimic the time for which processed food can remain in the colon before it is removed from the human body. The 2 h time was chosen to mimic the interaction of test compounds (orally administered) in an empty/partially filled stomach state. The 24 h time point was chosen because it is the approximate time of orally administered test compounds for interaction with the intestinal mucosa [26,27]. Colon biopsy punches were exposed to three different concentrations of graphene (1,10, and 100 µg/mL). Gene expression analysis was coupled with IPA to identify the affected pathways upon exposure to graphene and to predict upstream regulators. Furthermore, a total of 27 cytokine/chemokines were measured at the protein level using the Bioplex platform (Bio-Rad, Hercules, CA, USA), which were then correlated with gene expression data.

### 2.1. Dose- and Time-Dependent Effect of Graphene Exposure on mRNA Expression of Genes in the Colon

**Figure 2:**
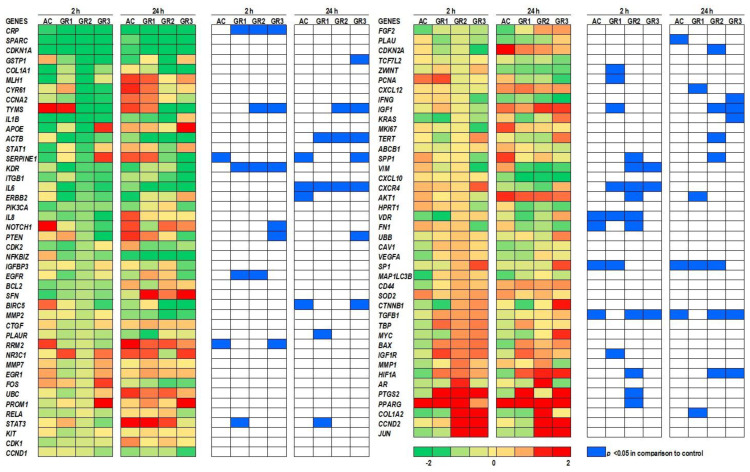
Heatmap analysis for genes that were differentially expressed after the exposure of colon tissue to graphene. Tissues were exposed to graphene at 1 (GR1), 10 (GR2), and 100 (GR3) µg/mL and to activated carbon (AC) at 100 µg/mL. Sampling was conducted at 2 and 24 h. Colors towards green indicate genes that were downregulated in relation to controls (water only). Colors towards red indicate upregulated genes in relation to controls (water only). Blue cells mark treatments with significant gene expression (*p* < 0.05) in comparison to controls (water only).

Taking into account both time points and all graphene concentrations tested, the gene expression analysis showed that out of the 86 total genes investigated in this study, 13 genes were significantly downregulated, and 24 genes were significantly upregulated after exposure to graphene (Figure 2).

Furthermore, we were able to identify certain genes that were significantly altered during the first 2 h of exposure to graphene and then returned to control levels. These genes included: *CRP, KDR, NOTCH1, EGFR, RRM2, ZWINT, PCNA, VIM, CXCR4, VDR, FN1, IGF1R, PTG85,* and *PPARG* (Table S1). In order to distinguish the different functional categories that could be involved in response to the early exposure of graphene, we performed Gene Ontology (GO) enrichment analysis using the PANTHER Classification System [28] to examine the functional distribution of the genes in our study. Results showed that molecular function binding (GO:0005488) exhibited significant enrichment among the genes analyzed in this group (Figure S1). In fact, graphene binding non-specifically to different proteins has been shown in several studies and in various tissues [29,30]. Among other significantly enriched molecular functions were the molecular transducer activity (GO:0060089) and molecular function regulator (GO:0098772) (Figure S1). In both categories, the *PCNA* (proliferating cell nuclear antigen) gene was shown to be highly upregulated in graphene-treated colon tissue during the first 2 h of exposure (Figure 2).

*PCNA* is an essential gene for eukaryotic cell proliferation [31,32]. Activation of this gene by graphene during early exposure could be an indication of the stimulatory effect of graphene toward eukaryotic cell proliferation. In fact, graphene has previously been used in 3D scaffolding to enhance the proliferation and differentiation of stem cells [33]. Moreover, *PCNA* is known to accelerate DNA polymerase in response to DNA damage. Our previous results showed that graphene causes cytotoxicity in macrophages, as measured by the release of lactate dehydrogenase (LDH) [24]. The damage graphene does to cells most likely causes the release of factors that induce the proliferation of new cells to replace them. In fact, PCNA expression in a biopsy from multiple myeloma patients was correlated with LDH release in the blood [34]. Apart from transient changes in gene expression (as described above), certain genes exhibited persistent significantly altered levels at 2 h and remained constant until 24 h. These genes included *TYMS, PTEN, SP1, TGFB1,* and *HIF1A* (Table S1). The consistent expression of *TYMS* (thymidylate synthase), which is involved in cell proliferation [35], along with *PTEN* (phosphatase and tensin homolog), which prevents cell growth and overly rapid proliferation [36], further confirms the interaction of pristine graphene with colon tissue and how the host mRNA gene expression plays a crucial role in the early stages of DNA biosynthesis [37]. Transcription of these genes could be regulated by transcription factor *Sp1* [38]. Another stimulatory gene was found among the listed genes known as *tgfb1* (transforming growth factor beta-1 proprotein). This gene exhibited a decrease in mRNA expression between 2 and 24 h. However, its expression remained significantly higher than the control (*p* < 0.05). The third category of genes included genes that were altered at 24 h and did not exhibit any changes during the first 2 h of exposure. These genes included *GSTP1, IL6, BIRC5, CDKN2A, CXCL12, IFNG, IGF1, KRAS, TERT, SPP1, AKT1,* and *COL1A2* (Table S1). The functional gene classification resulted in three molecular function categories, which included binding (GO:0005488), catalytic activity (GO:0003824), and molecular function regulator (GO:0098772) (Figure S1). The gene regulation differences over the 24 h period was indicative of the activation of different pathways in response to graphene exposure. The activation of genes in response to pristine graphene exposure was triggered by the nanoparticle’s characteristics of the material rather than being a carbon allotrope. Activated carbon, another carbon allotrope but not in a nano size, was used as a negative control and has shown a different gene expression response in comparison to pristine graphene (Figure 2). In fact, 6 genes out of 86 genes have shown significant differences in gene expression compared to controls during the first 2 h of exposure, and 7 genes during the 24 h of exposure. The gene expression was reversed in the case of many genes, including *TGFB1, SP1,* and *VDR*, where the expression was downregulated in activated-carbon-treated tissue and upregulated in the graphene-treated samples.

Binding or adhesion of graphene to the surface of colonic cells was detected at 2 h and sustained a gene expression response for key genes including *KRAS, TERT, SPP1, BIRC5,* and *TGFB1*. In our study, the binding or adhesion of graphene to epithelial or immune cells could have led to the downstream modulation of molecular and catalytic activity, as shown in an earlier study [39]. Furthermore, hydrophobic materials are more likely to adhere to the lipid bilayers of cell membranes [40,41]. For that reason, pristine graphene is more prone to adhere to cell surfaces than other functionalized graphene such as graphene oxide [39]. On the other hand, it is possible that pristine graphene can bind to bacteria residing on the intestinal mucosal surface/villi and alter the interaction of the microbial population with intestinal epithelial cells. In fact, Tu et al. showed that pristine graphene nanosheets induced degradation in the outer and inner membranes of *E. coli* and reduced their viability [42]. Thus, the initial binding of pristine graphene could modulate the signaling pathways in epithelial and immune cells. Real-time PCR data were further investigated with IPA to understand the pathways impacted by graphene exposure.

### 2.2. The Canonical Pathways and Diseases Affected by Graphene Exposure

The fold change and *p*-values obtained following the raw data gene expression analysis were then mechanistically investigated using IPA software (Qiagen). Pathway analysis was then carried out with 86 genes that fulfilled the threshold cut-off of -log2 fold change and *p*  <  0.05 in the graphene-treated colon tissue and compared to the control non-treated colon tissue. Canonical pathways that had activation z-score values were taken for further analysis and pathways that showed no activity patterns were omitted.

**Figure 3:**
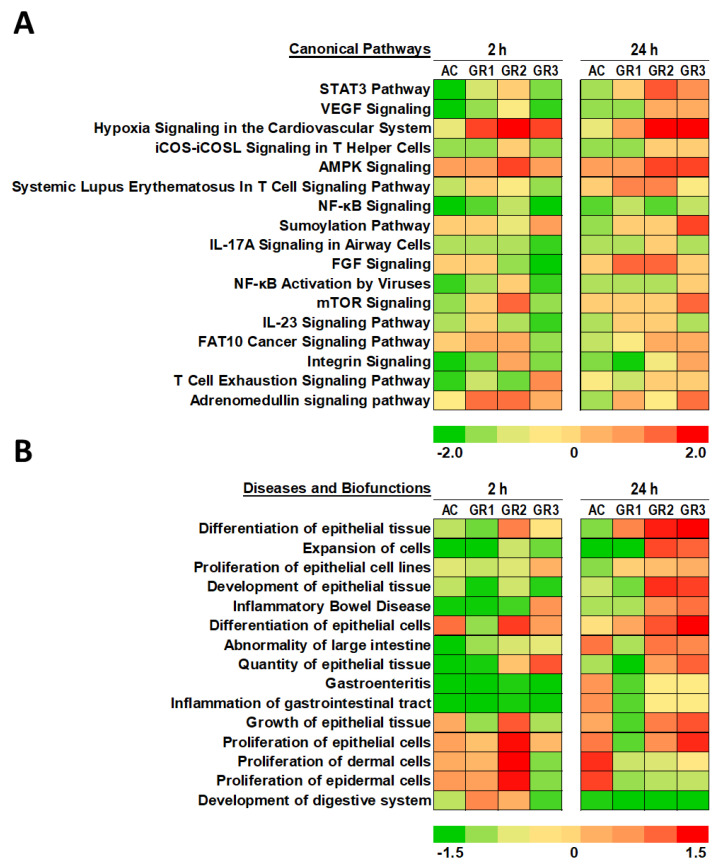
Heatmap analysis of (**A**) canonical pathways and (**B**) diseases and biofunctions affected by graphene exposure to colon tissue. Tissue was exposed to graphene at 1 (GR1), 10 (GR2), and 100 (GR3) µg/mL and to activated carbon (AC) at 100 µg/mL. Sampling was conducted at 2 and 24 h. Colors toward green indicate functions with low z-scores. Colors toward red indicate functions or pathways with higher z-scores.

The STAT3 (signal transducer and activator of transcription 3) signaling pathway (-log *p* = 14.348) and VEGF (vascular endothelial growth factor) signaling pathways (-log *p* = 9.692) were the most significant canonical pathways. Both signaling pathways showed higher z-scores. The STAT3 pathway in colon tissue treated with graphene at 10 µg/mL showed the highest positive activation z-score of 1.15 among other graphene doses, indicating overall upregulation of the pathway, whereas the highest dose of graphene at 100 µg/mL showed a lower z-score of 0.577, indicating upregulation of the pathway compared to controls (Figure 3A and Figure S2). The VEGF signaling pathway showed a positive z-score of 0.33 in both doses 10 and 100 µg/mL of graphene after 24 h exposure.

STAT3 is a member of the STAT protein family, which exerts a signal transducer in the cytoplasm and a transcription activator in the nucleus. It is usually activated by cytokines and growth factors. [43]. Vascular endothelial growth factor can induce cellular processes which are common to many growth factor receptors, including cell migration, proliferation and survival. The activation of both pathways is indicative of an increase in cell proliferation after exposure to graphene at all doses of pristine graphene. This finding is in concert with the finding of Liu et al., who reported an increase in cell proliferation and cell growth in numerous cell lines (MCF-7, HepG2, A549 and HeLa Cells) when they were treated with pristine graphene at different doses [44]. Moreover, graphene induced cell proliferation in normal cells as well [44].

The activation of cell proliferation in colonic tissue after exposure to graphene could be linked to alteration of the immune response. In fact, IL-23, and IL-17 signaling pathways have appeared as the major pathways affected by colonic tissue after graphene exposure (Figure 3A). It is known that IL-23 could lead to the activation of STAT3, and consequently increase the levels of IL-17 [45]. IL-17 activates NF-κB and stimulates the production of several inflammatory mediators, including IL-6, IL-8, and GM-CSF [46].

During the analysis of diseases and biofunctions affected by graphene exposure, we filtered data to highlight only biofunctions related to the gastrointestinal system. Figure 3B shows the heatmap of z-scores for all 15 biofunctions affected by graphene after 2 and 24 h exposure. Most biofunctions analyzed belonged to the categories of epithelial cell expansion, development, and proliferation. Indeed, differentiation of epithelial tissue function was the highest significant biofunction, with −log *p* = 40.2. Colon tissue exposed to graphene for 24 h and at different doses exhibited a dose-dependent increase in z-score, which indicates upregulation of this biofunction. In fact, colon tissue exposed to graphene at 1, 10, and 100 µg/mL showed z-scores of 0.5, 1.287, and 1.704, respectively. Certain biofunctions were found to be highly upregulated during the first 2 h of exposure and remained significantly high until 24 h of graphene exposure. Colon tissue exposed to graphene at 10 µg/mL is a good example of how four biofunctions (differentiation of epithelial cells, quantity of epithelial tissue, growth of epithelial tissue, and proliferation of epithelial tissues) showed early (2 h) and lasting activation (24 h) of different biofunctions (Figure 3B). Most of these genes indicate prolonged cell stimulation by graphene exposure, which could lead to the observed proliferation and differentiation of epithelial cells. Moreover, out of all the biofunctions analyzed using IPA, two biofunctions (gastroenteritis and inflammation of gastrointestinal tract) showed negative z-scores at the 2 and 24 h time points in comparison to controls, which indicates downregulation of these functions.

### 2.3. Upstream Regulators Based on IPA Analysis

Upstream regulator analysis was performed to identify regulators that may be responsible for the observed changes in gene expression. Based on the z-score algorithm, IPA predicts which upstream regulators are likely to be activated or inhibited, which, in turn, can explain the gene expression changes observed in a dataset [47].The z-score value was calculated using gene expression patterns of the genes downstream of an upstream regulator. The *p*-value of overlap indicated the statistical significance of genes in the dataset that were downstream of the upstream regulator, but unlike the z-score, it does not take the upregulation or downregulation of genes in a dataset into consideration. Upstream regulators with a z-score greater than 1 or less than -1 and a *p*-value of 0.05 were considered significant, and their roles as regulators were further studied.

**Figure 4:**
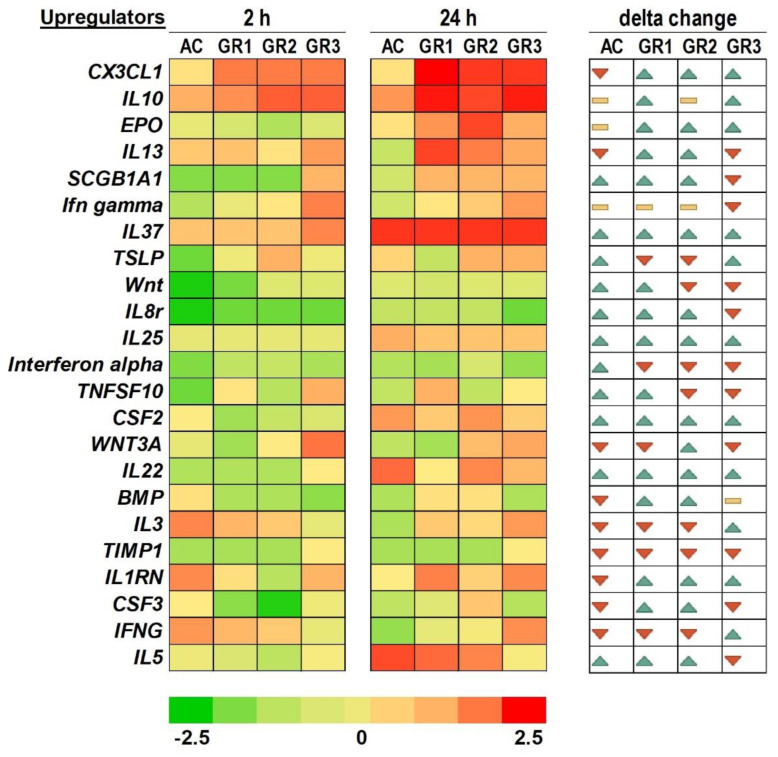
Heatmap analysis of upstream regulators of genes affected by graphene exposure to colon tissue. Tissue was exposed to graphene at 1 (GR1), 10 (GR2), and 100 (GR3) µg/mL and to activated carbon (AC) at 100 µg/mL. Sampling was conducted at 2 and 24 h. Colors toward green indicate functions with low z-scores. Colors toward red indicate functions or pathways with higher z-scores. Changes in shades from lighter to darker indicate lower to higher changes. Delta changes between 2 and 24 h time points are indicated by increases (green arrow), decreases (red arrow) or no change (yellow bars).

This analysis revealed that several upstream regulators are involved in controlling the differential expression of genes due to graphene treatment. These regulators and their activation z-scores are shown in the heatmap in Figure 4. The list of upstream regulators was filtered to include upstream regulators that have immune functions. A total of 23 upstream regulators were found and can be categorized into three groups: (a) genes that were predicted to increase from 2 to 24 h after graphene treatment independent of dose, including CX3CL1, EPO, IL-37, IL-25, CSF2, IL-22, and IL-1RN; (b) genes that were predicted to decrease from 2 to 24 h independent of graphene doses, including interferon alpha and TIMP1; and (c) genes that showed changes in predicted expression between 2 and 24 h, including the 14 remaining genes. In these upstream regulators, we found that their magnitude of expression was opposite at the highest concentration (100 µg/mL) in comparison to the other two concentrations (1 and 10 µg/mL).

### 2.4. Immune Response after Exposure to Graphene

**Figure 5:**
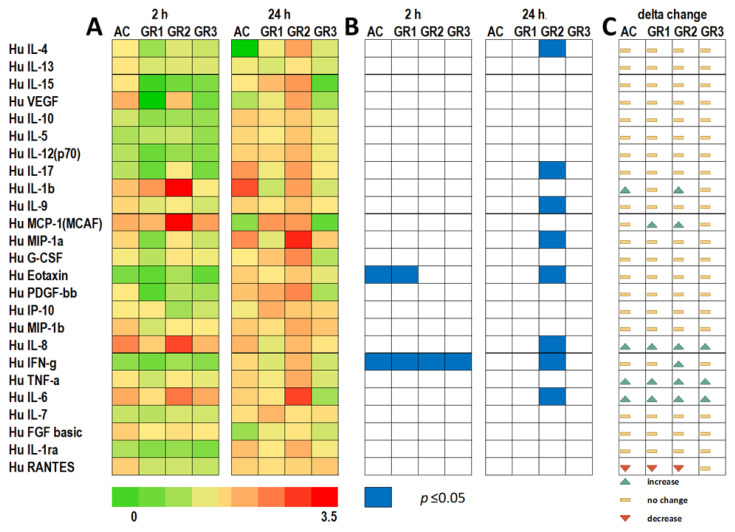
Heatmap analysis for proteins that were differentially expressed after the exposure of colon tissue to graphene. Tissues were exposed to graphene at 1 (GR1), 10 (GR2), and 100 (GR3) µg/mL and to activated carbon (AC) at 100 µg/mL. Sampling was conducted at 2 and 24 h. (**A**) Colors toward green indicate proteins that were downregulated in relation to control (water only). Colors toward red indicate upregulated genes in relation to control (water only). (**B**) Blue cells mark treatments that have significant proteins levels (*p* < 0.05) in comparison to control (water only). (**C**) Delta changes in protein levels between the 2 and 24 h time points are indicated by increases (green arrow), decreases (red arrow), or no change (yellow bars).

To further confirm the immune response of human colonic tissue to graphene exposure, total protein was extracted from tissue at 2 and 24 h exposure, and levels of cytokines were assessed using a 27-plex Bioplex Multiplex Immunoassay System. A heatmap based on actual values of observed concentrations of cytokines produced is shown in Figure S3. The results showed that two cytokines had values outside of the detectable range (IL-2 and GM-CSF). Remaining cytokines had observed concentrations ranging from 0.311 ng/µL (IL-4) to 2704 ng/µL (IL-6). Results of fold changes for 23 different cytokines in comparison to controls are summarized in a heatmap (Figure 5A). The comparison of observed cytokine concentrations in the colon between the 2 and 24 h time points showed that the overall production of cytokines did not change for 14 out of 23 cytokines (Figure 5B). Moreover, we found that six cytokines, including IL-1β, MCP-1, IL-8, IFN-γ, TNF-α, and IL-6, showed significant increases at the 24 h time point in comparison to the 2 h time point (Figure 5C). RANTES was the only cytokine that showed a decrease in cytokine levels between the 2 and 24 h time points. Furthermore, seven different cytokines showed significant fold changes in comparison to the control (Figure S4). These cytokines included IL-17, IL-9, MIP1α, Eotaxin, IL-8, IFN-γ and IL-6. The latter cytokines and chemokines are part of the pro-inflammatory response which is usually produced by macrophages. IL-17 communicates with Jak-STAT family signaling, particularly STAT3. IL-17, IL-6, and IL-8 showed positive associations with VEGF expression and signaling [48]. The IL-17–IL-23 cytokine signaling axis has been identified as a tumor-promoting pathway for “inflammation-associated and sporadic cancers”, of several organs including the colon [49]. The IL-23 cytokine was not present in the cytokine array used in this study; nonetheless, mRNA expression of IL-23 was significantly higher upon graphene exposure. Thus, the comprehensive results of our study at the mRNA expression level (IL-23) and the protein level (IL-17) clearly indicate the initiation of cell proliferation in colon tissue.

Interestingly, these findings also correlate with our earlier findings, where we studied the impact of graphene on the intestinal microbiota. In fact, an increased abundance of butyrate-producing bacteria was noticed [21]. Butyric acid is known to positively modulate the immune system and induce epithelial cell proliferation [50,51]. Thus, it is plausible that graphene perturbation to the intestinal mucosa (either via direct interaction with intestinal epithelial cells or via interaction with the intestinal mucosa-associated bacterial species) could have consequential impacts on the host immune system.

## 3. Materials and Methods

### 3.1. Characterization of Pristine Graphene

Pristine Graphene (1–1.2 nm thick, ≤10 μm lateral dimensions) was characterized at the Nanocore facility, National Center for Toxicological Research, Jefferson, Arkansas, and at the Center of Integrative Nanotechnology Sciences, University of Arkansas at Little Rock, Arkansas, as previously described [24,52]. Before use, pristine graphene was subjected to three autoclavations (121 °C, 30 min, 15 psi) to remove any endotoxin contamination [24] and then suspended in 0.5% BSA. Graphene was then sonicated in a water bath (Branson Ultrasonics, Fremont, CA, USA) at 25 °C and 40 kHz for 15 min for improved nanoparticle dispersion.

### 3.2. Exposure of Human Colon to Pristine Graphene

The use of colon tissue samples was reviewed and approved by the U.S. Food and Drug Administration’s Research Involving Human Subject Committee (RIHSC #12-062T exemption). The inclusion and/or exclusion criteria for the human subjects included in this study were that the colon samples should be from de-identified subjects aged 18 years or older who do not have inflammatory bowel disease or pathological conditions involving the colon. Colon tissues from three subjects (two male and one female, with age range from 35 to 60 years) were obtained from the Cooperative Human Tissue Network—Southern Division. Sectioned intestinal tissues were shipped in RPMI media with overnight delivery. Upon receipt, tissues were immediately transferred into a cocktail of 50% MACS tissue storage solution (Miltenyi Biotec, Bergisch Gladbach, Germany) and 50% RPMI-1640 (ATCC, Manassas, VA, USA) media containing 1% fetal bovine serum and 5% streptomycin/penicillin (ATCC; Manassas, VA, USA). Tissue biopsy punches 6.00 mm in size were collected and placed into the upper compartment of transwells (Corning Life Sciences, Union City, CA, USA) filled with 200 µL of growth media, as described previously [53]. The lower compartment of each transwell was filled with 1.0 mL of the same medium. Tissues were incubated at 5% CO_2_ and 37 °C for 2 h. Next, pristine graphene nanoparticles were added to the upper compartment at final concentrations of 1, 10, or 100 µg/mL. The selection of concentration was based on the risk of low versus high concentration plus a medium concentration, as defined elsewhere [24,54]. A negative control of activated carbon (AC) at 100 µg/mL was also used. All tissues were collected at 2 and 24 h after incubation and stored at −80 °C for further analysis.

### 3.3. Transcription and Quantitative Real-Time Polymerase Chain Reaction (qRT-PCR) Assay

The total RNA of colonic tissues was extracted using an RNeasy Mini kit (Qiagen, Valencia, CA, USA). Obtained RNA was treated with DNAse. This DNAse-treated RNA was reverse-transcribed using a PrimeScript ™ RT reagent kit (Takara, Otsu, Shiga, Japan). After cDNA synthesis, real-time PCR was performed on PrimePCR plate Intestinal neoplasm Tier 1 apparatus using SYBR green to amplify PCR products. Real-time PCR was conducted at the optimized annealing temperature of 60 °C, as described previously [22]. The relative quantification of targeted genes in comparison to a reference GADPH gene was determined. Final results were expressed as a relative expression ratio between targeted genes and the reference gene.

### 3.4. Ingenuity Pathway Analysis

Data generated by real-time PCR (fold change and *p* value; as compared to control) were further analyzed by Ingenuity Pathway Analysis (IPA; v8.0, Qiagen, Valencia, CA, USA) software to identify the pathways and functions that were significantly affected due to graphene exposure The IPA software analyzed the real-time gene expression data based on algorithm predictions, providing a list of potential up regulators organized based on their z-scores (significance). A detailed description of IPA is available on the Ingenuity Systems website (http://www.ingenuity.com, accessed on 1 May 2021). It calculates a probability value for each pathway/function according to the fit of users’ data to the IPA database using the right-tailed Fisher’s exact test. Pathways with *p* < 0.05 were considered significantly affected.

### 3.5. Multiplex Cytokines Assay

Gut-associated mucosal chemokine and cytokine levels were evaluated in the colon tissue lysate using a Bioplex human 27-plex panel following the manufacturer’s instructions. This technology represents a powerful tool in the quantitation of several cytokines simultaneously, with a higher dynamic range as compared to ELISA [55]. Colon tissue protein extraction was performed by the addition of 100 µL of lysis solution (Bio-Rad, Hercules, CA, USA) per 10 mg of colon tissue. Samples were homogenized using a gentle MACS dissociator (Miltenyi Biotec, Inc., Auburn, CA, USA) in the intestinal tissue setting. The lysate was centrifuged at 4 °C for 10 min at 1000 rpm, and the clear homogenate was transferred to 1.5 mL Eppendorf tubes and centrifuged at 4 °C for 15 min at 12,000 rpm. The clear supernatant was transferred into new 1.5 mL Eppendorf tubes, diluted, and the protein concentration was measured using the Bradford Assay (Bio-Rad, Hercules, CA, USA). Samples were then stored at −80 °C until further use.

### 3.6. Statistics

All data are presented as mean values ± SE (standard errors). Statistical analysis was conducted using SPSS^®^ software; Chicago, IL, USA by performing repeated-measures ANOVA for time-effect analysis and ANOVA with post hoc analysis using the Tukey test for treatment differences. Statistical significance was determined by *p* < 0.05.

## 4. Conclusions

**Figure 6:**
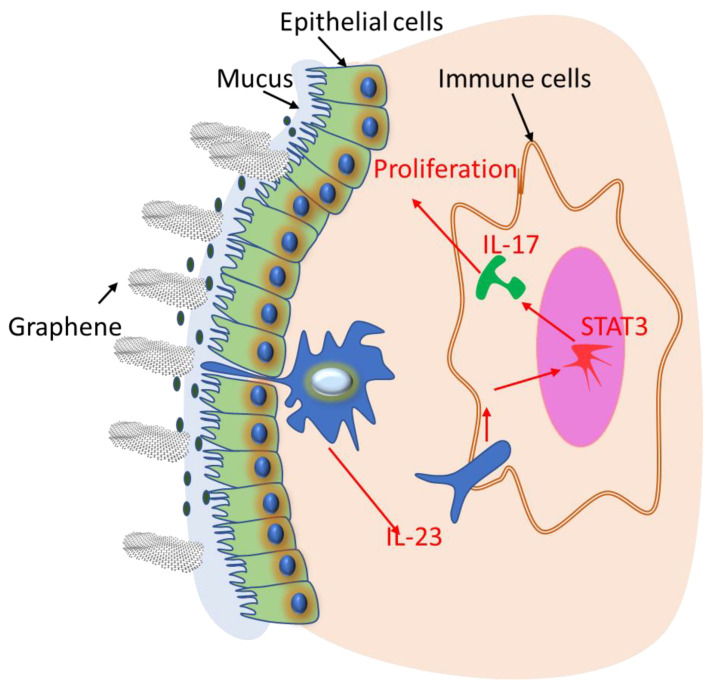
Schematic figure of the interaction of graphene with cells of human colonic tissue.

This study was conducted to address the knowledge gap related to the risk/safety assessment during the interaction of pristine graphene with the human intestinal barrier using an ex vivo model (a human relevant translation model). Our results highlighted the pathways affected by graphene upon tissue exposure. We have shown that graphene can stimulate the mRNA expression of genes involved in cell proliferation and growth upon binding/adhering to epithelial tissue. This interaction is coupled with the dose-dependent activation of a pro-inflammatory response through many pathways. The correlation between real-time PCR and protein data showed that the IL-23–IL-17 axis signaling and its interaction with STAT3 emerged as principal pathways by which graphene could impact human epithelial tissue (Figure 6). Additional investigations on the subtypes of cells responsible for the observed biological effect will be necessary to fully understand the toxicity and long-term impact of pristine graphene.

## Data Availability

The data presented in this study are available on request from the corresponding author. The data are not publicly available due to privacy reasons.

## Supplementary Materials

The following are available online at https://www.mdpi.com/article/10.3390/ijms222111443/s1.

## Abbreviations

AC	Activated carbon;
GR	Graphene;
GRMs	Graphene-related materials;
GO	Graphene oxide;
IPA	Ingenuity Pathway Analysis;
PCNA	Proliferating cell nuclear antigen;
TGFB1	Transforming growth factor beta-1;
STAT3	Signal transducer and activator of transcription 3;
VEGF	Vascular endothelial growth factor.
