# Supplementary material for: Ex Vivo Human Colon Tissue Exposure to Pristine Graphene Activates Genes Involved in the Binding, Adhesion and Proliferation of Epithelial Cells
Source: Int J Mol Sci. 2021 Oct 23;22(21):11443. doi: 10.3390/ijms222111443 (PMC8584180; doi:10.3390/ijms222111443)
Supplement: Supplementary file 1 [file ijms-22-11443-s001.zip › ijms-1420318-supplementary.pdf]

Supplementary files

Table S1. List of gene symbols and their corresponding gene names. This list includes only the genes that have shown significant differences in the analysis of gene expression data.

Gene Symbol	Gene Name
CXCL12	chemokine (C-X-C motif) ligand 12
PTEN	phosphatase and tensin homolog
IL6	interleukin 6 (interferon, beta 2)
PTGS2	prostaglandin-endoperoxide synthase 2 (prostaglandin G/H synthase and cyclooxygenase)
SPP1	secreted phosphoprotein 1
PCNA	proliferating cell nuclear antigen
AKT1	v-akt murine thymoma viral oncogene homolog 1
HIF1A	hypoxia inducible factor 1, alpha subunit (basic helix-loop-helix transcription factor)
COL1A2	collagen, type I, alpha 2
CRP	C-reactive protein, pentraxin-related
IFNG	interferon, gamma
IGF1	insulin-like growth factor 1 (somatomedin C)
KDR	kinase insert domain receptor (a type III receptor tyrosine kinase)
IGF1R	insulin-like growth factor 1 receptor
PPARG	peroxisome proliferator-activated receptor gamma
VIM	vimentin
KRAS	v-Ki-ras2 Kirsten rat sarcoma viral oncogene homolog
SP1	Sp1 transcription factor
BIRC5	baculoviral IAP repeat containing 5
NOTCH1	notch 1
CDKN2A	cyclin-dependent kinase inhibitor 2A (melanoma, p16, inhibits CDK4)
TYMS	thymidylate synthetase
GSTP1	glutathione S-transferase pi 1
EGFR	epidermal growth factor receptor
RRM2	ribonucleotide reductase M2
VDR	vitamin D (1,25- dihydroxyvitamin D3) receptor
TERT	telomerase reverse transcriptase
TGFB1	transforming growth factor, beta 1
FN1	fibronectin 1

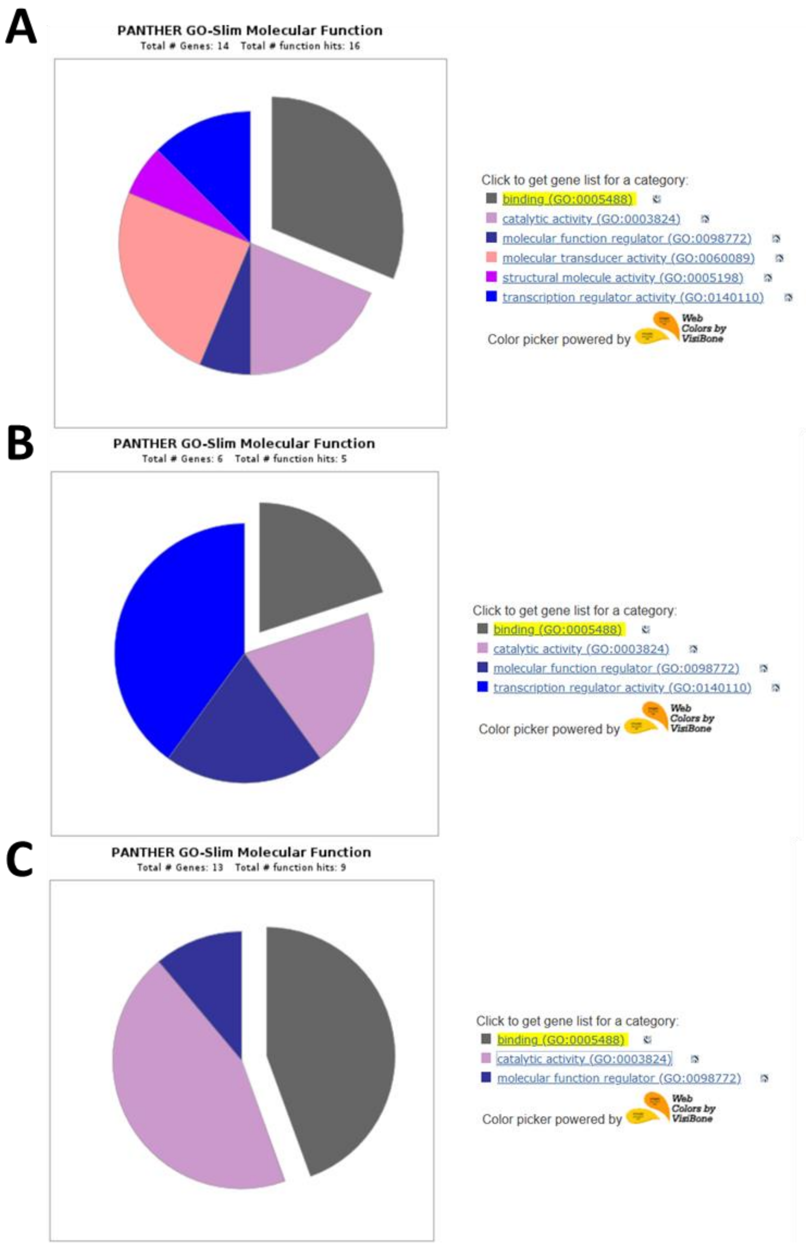

Figure S1. Molecular function categories of genes significantly affected by graphene. (A) genes that were significantly altered during the first 2 hrs of exposure to graphene and then returned to control levels. (B) genes that exhibited significantly altered levels at 2 hrs and remained constant until 24 hrs. (C) genes that were altered at 24 hrs and did not show any changes during the first 2 hrs of sampling.

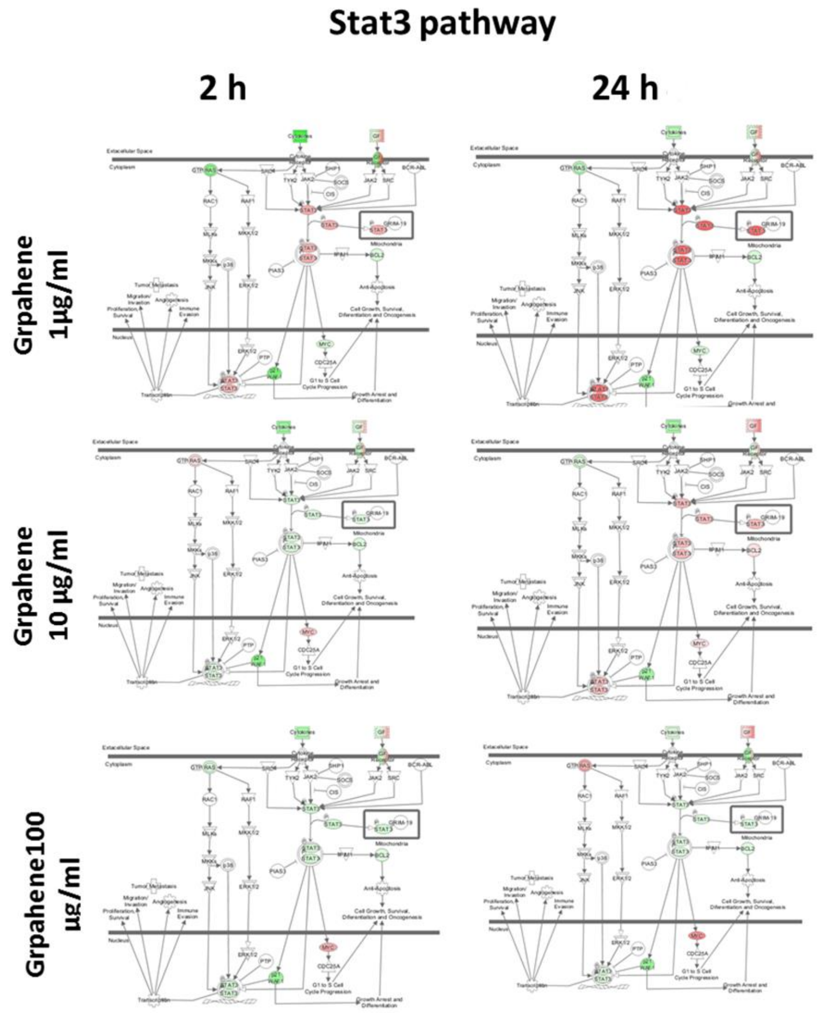

Figure S2. STAT3 pathway analysis after exposure to graphene at three different concentration at the 2 and 24 hrs time points.

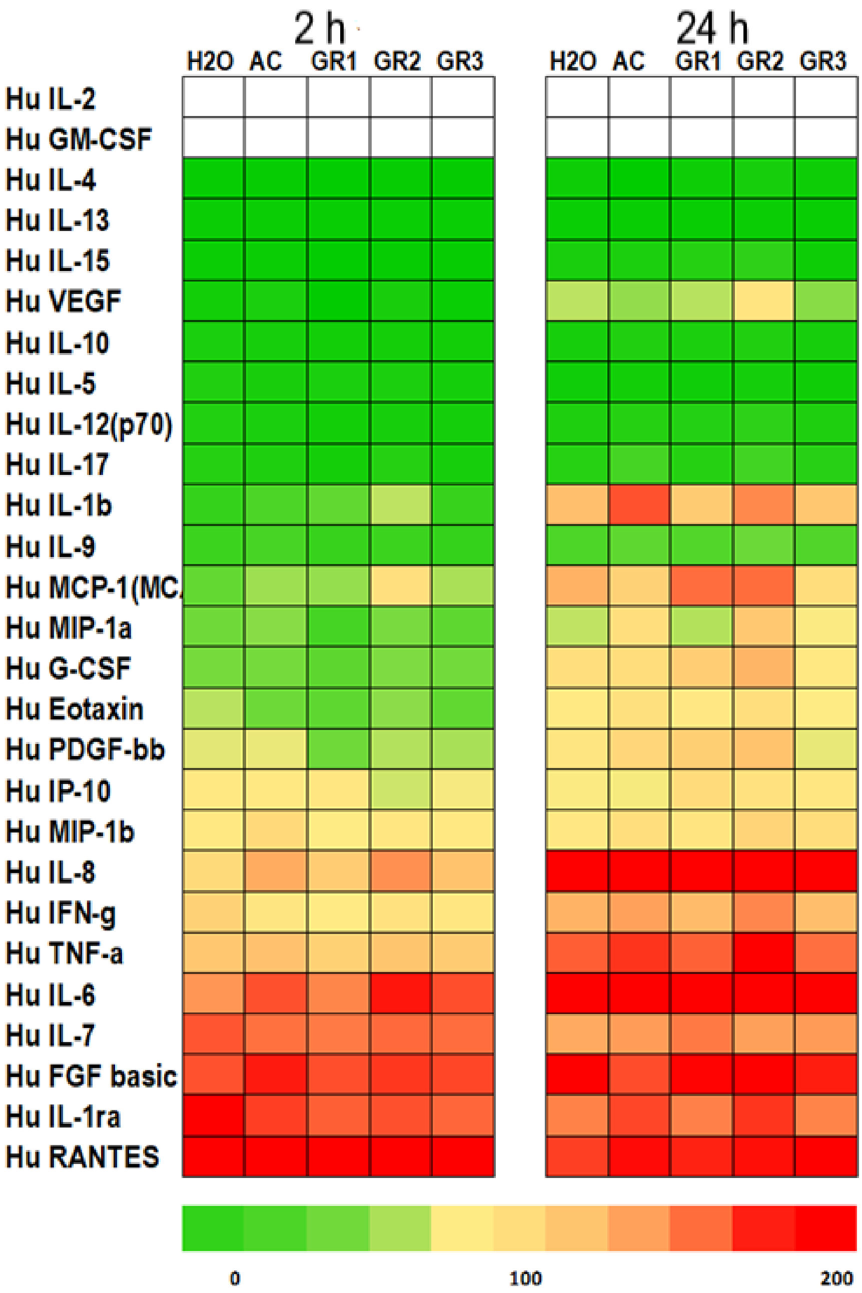

Figure S3. Heatmap of 27-plex Bioplex data analysis. Tissue was exposed to graphene at 1 (GR1), 10 (GR2) and 100 (GR3) μg/mL and to activated carbon (AC) at 100 μg/mL. Sampling was conducted at 2 and 24 hrs. Colors toward green show proteins with low levels and colors toward red show proteins at higher levels.

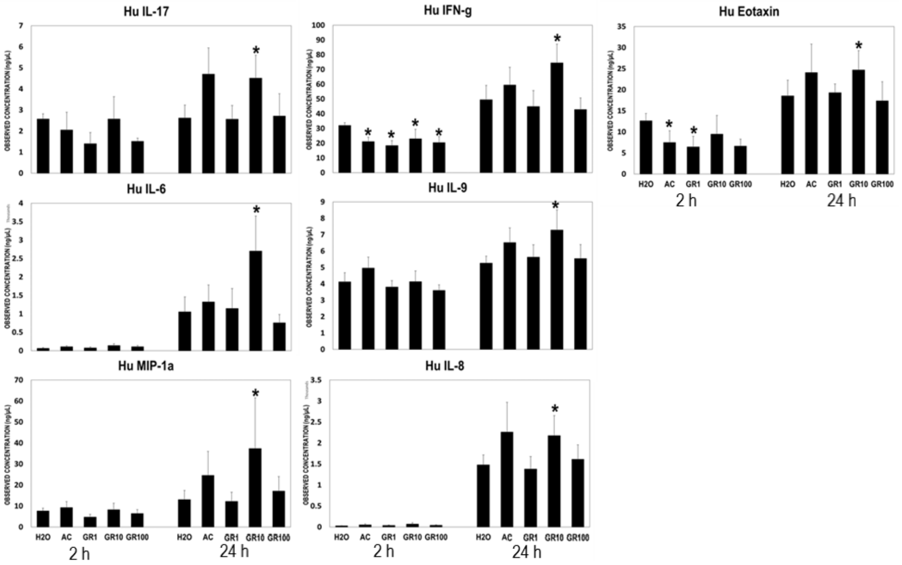

Figure S4. Observed concentrations of proteins that showed significant levels change in comparison to control (water only). Tissue was exposed to graphene at 1 (GR1), 10 (GR2) and 100 (GR3) μg/mL and to activated carbon (AC) at 100 μg/mL. Sampling was conducted at 2 and 24 hrs. (*) treatment that showed significant difference in comparison to corresponding control (p<0.05)
